# Simultaneous coronary thrombosis with multisite myocardial infarction and complex malignant arrhythmia: A case report

**DOI:** 10.1097/MD.0000000000020994

**Published:** 2020-07-02

**Authors:** Mu-li Wu, Duan-Min Xu, Chang Chen, Ye-qun Chen, Yi-Fan Sun, Chuang-jia Hu

**Affiliations:** Department of Cardiology, First Affiliated Hospital of Shantou University Medical College, Shantou, China.

**Keywords:** acute myocardial infarction, malignant arrhythmia, simultaneous coronary thrombosis

## Abstract

**Introduction:**

Acute myocardial infarction with simultaneous coronary thrombosis has been rarely reported. This combination induces various arrhythmias and is a high-risk factor for cardiogenic shock.

**Patient concerns:**

A 65-year-old man presented with sweating and a 3-h abrupt persistent back pain that radiated to the anterior.

**Diagnosis:**

Multisite myocardial infarction, coronary thrombosis with and complex malignant arrhythmia

**Interventions:**

Prompt intervention includes cardiac pacing, percutaneous coronary intervention (PCI), thrombus aspiration and intra-aortic balloon pump (IABP).

**Outcomes:**

The patient was successfully rescued after PCI and thrombus aspiration.

**Conclusions:**

Recognition of dynamic electrocardiographic changes enhances our understanding of the pathogenesis of myocardial infarction.

## 1 Introduction

Acute myocardial infarction is believed to underlie ∼75% of deaths in patients with sudden cardiac death and is the most common cause of death worldwide.^[1]^ Acute myocardial infarction usually results from a blockage in one or more of the coronary arteries. Reperfusion therapy through percutaneous coronary intervention (PCI) for acute ST-elevation myocardial infarction is the primary strategy for prompt restoration of coronary circulation.^[2,3]^ Reperfusion reduces infarct size and improves residual cardiac function after acute myocardial infarction.^[2]^ Simultaneous thrombosis of multiple coronary arteries in patients with acute myocardial infarction has been rarely reported.^[4]^ However, this combination is associated with a high-risk of cardiogenic shock and life-threatening arrhythmia.^[5–7]^ It is of clinical importance to properly diagnose this condition to begin effective treatment. Herein, we report a case of a 65-year-old man suffering from simultaneous anterior and inferior myocardial infarction due to stenotic and thrombotic occlusion in the left anterior descending artery (LAD) and distal circumflex artery (LCX).

## 2 Case report

**Figure 1:**
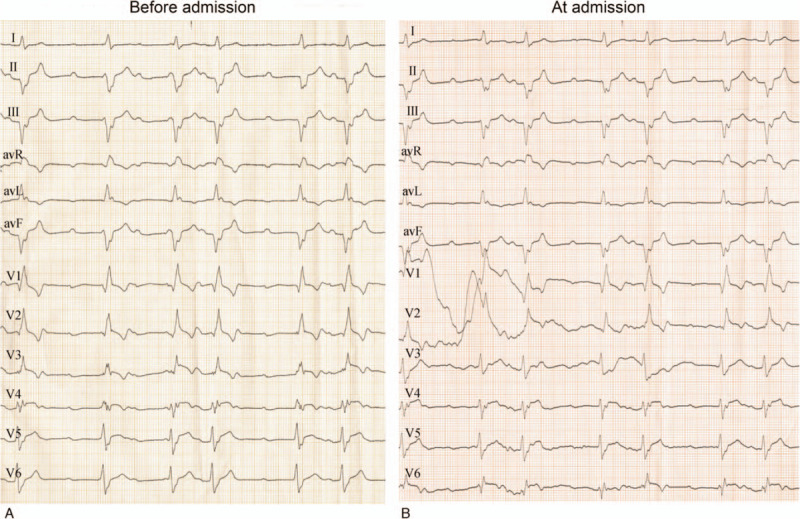
(A) Electrocardiography (ECG) performed in a local hospital before admission indicates sinus tachycardia, high atrioventricular block (3:1–4:1), junctional escape rhythm, complete right bundle branch block (RBBB), and inferior and anterior myocardial infarction. (B) ECG performed in the Emergency Department at admission displays sinus tachycardia, high atrioventricular block (3:1–4:1), junctional escape rhythm, complete RBBB, left anterior fascicular block (LAFB), and anterior myocardial infarction.

A 65-year-old man presented to the emergency room with sweating and a 3-h abrupt persistent back pain that radiated to the anterior. The patient reported a 10-year smoking history but no other additional medical history. Earlier electrocardiography (ECG) at a local hospital did not show typical signs of myocardial infarction (Fig. 1A). A successful cardiopulmonary resuscitation was achieved following a sudden cardiac arrest at admission. ECG performed when the patient was admitted to the Emergency Department indicated sinus tachycardia, high atrioventricular block (3:1–4:1), left anterior fascicular block (LAFB), complete right bundle branch block (CRBBB) (Fig. 1B). There was a noticeable wave transformation from QS to rS in the II, III, and avF, and no obvious ST elevation and depression in all leads in the ECG at admission. Biochemical tests showed that there were elevated troponin T (TnT) levels of <40 ng/L and a D-dimer of 0.55 μg/mL.

**Figure 2:**
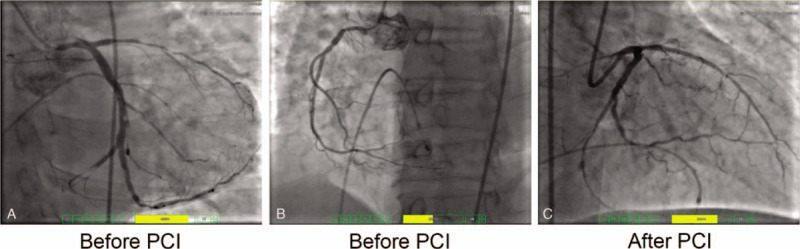
Emergency coronary angiography after cardiac pacing showed (A) Before PCI, CAU (caudal) 30° position: complete occlusion at the beginning of the middle segment of the anterior descending artery (LAD) with TIMI grade 0; 99% stenosis caused by thromboembolism at the distal circumflex artery (LCX) with TIMI grade 2; 90% stenosis at the third left obtuse marginal branch with TIMI grade 3. (B) Before PCI, left anterior oblique (LAO) 30° + CAU 30° position: 70% stenosis at the proximal right coronary artery. (C) After PCI, right anterior oblique (RAO) 27° + cranial (CRA) 31° position: a Helios 2.75∗22 mm stent was implanted in the proximal middle segment of the anterior descending artery, and the blood flow of the anterior descending artery was restored to TIMI grade 3. Percutaneous transluminal coronary angioplasty was performed in the distal circumflex artery after removing thrombosis and the blood flow was restored to TIMI grade 3.

Since the patient quickly presented with unstable signs of systolic blood pressure (SBP) 80 mm Hg), heart rate (HR) 40 beats per minute (bpm), and shortness of breath, cardiac pacing and subsequent emergent coronary angiography (CAG) were performed. We found a complete occlusion in the proximal LAD artery with TIMI grade 0, a thrombosis which caused a nearly complete stenosis (99%) in the distal LCX with TIMI grade 2, and 70% stenosis in the proximal right coronary artery (RCA) (A and B). A coronary stent was implanted in the LAD after temporary cardiac pacing. The thrombosis in the distal LCX was removed by aspiration and percutaneous transluminal coronary angioplasty (PTCA) was performed (Fig. 2C).

**Figure 3:**
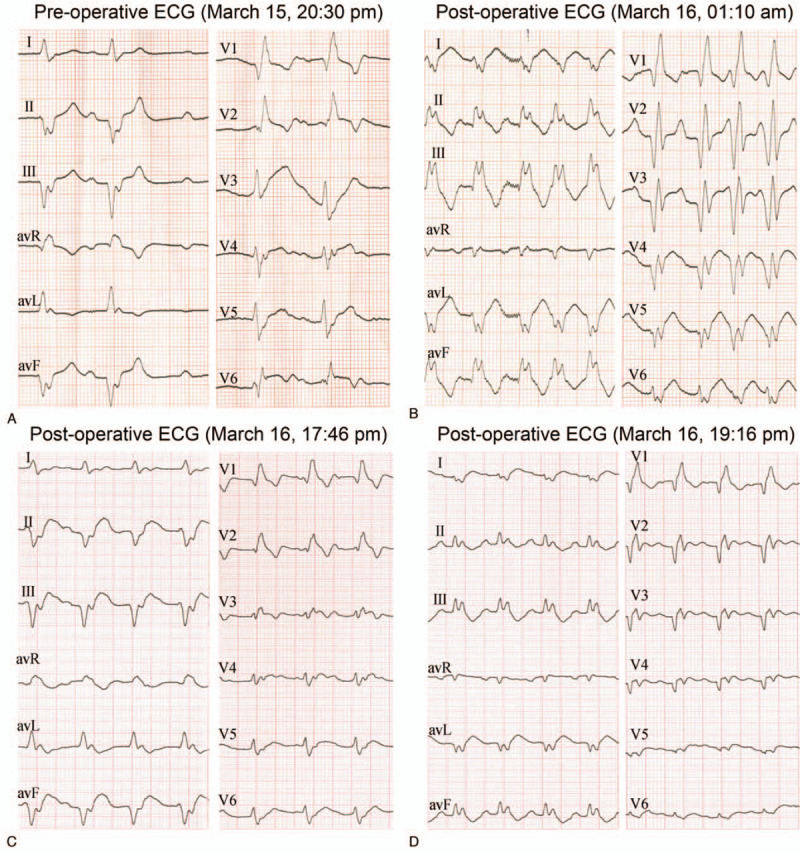
Postoperative echocardiography (ECG) showed regressed left anterior fascicular block (LAFB) and extensive anterior Q wave. (A) The pre-operative ECG at ER (time: March 15, 20:30 p.m.) showed LAFB without extensive anterior Q waves. (B) The second ECG (time: the 1st day after the operation; 01:10 a.m.) showed an absence of LAFB, a complete left posterior branch block with extensive anterior Q waves. (C) The third ECG (time: the 1st day after the operation; 17:46 p.m.) displayed a reappearance of LAFB with disappearing anterior Q waves. (D) The fourth ECG (time: the 1st day after the operation; 19:16 p.m.) showed a disappearing LAFB and extensive anterior Q waves reappeared.

Cardiogenic shock appeared during the operation. Intra-aortic balloon pump (IABP) was employed and the patient returned to the ward with improved symptoms. Interestingly, the evolution of ECG changes before and after the operation could be characterized into two groups. First, preoperative ECG showing no ST segment changes and pathological Q waves in the anterior and inferior leads turned into pathological Q waves in the extensive anterior wall after the operation. Second, dynamic ECG changes appeared postoperatively. Extensive anterior Q waves reappeared periodically with the disappearance of left anterior fascicular block (LAFB). Postoperative ECG showed sinus tachycardia, first-degree atrioventricular block, CRBBB, and acute extensive anterior myocardial infarction. Interestingly, a regressed LAFB and extensive anterior Q wave were detected (Fig. 3). The appearance of LAFB on the ECG was accompanied by absent anterior Q waves, thus masking the manifestation of extensive anterior myocardial infarction.

**Figure 4:**
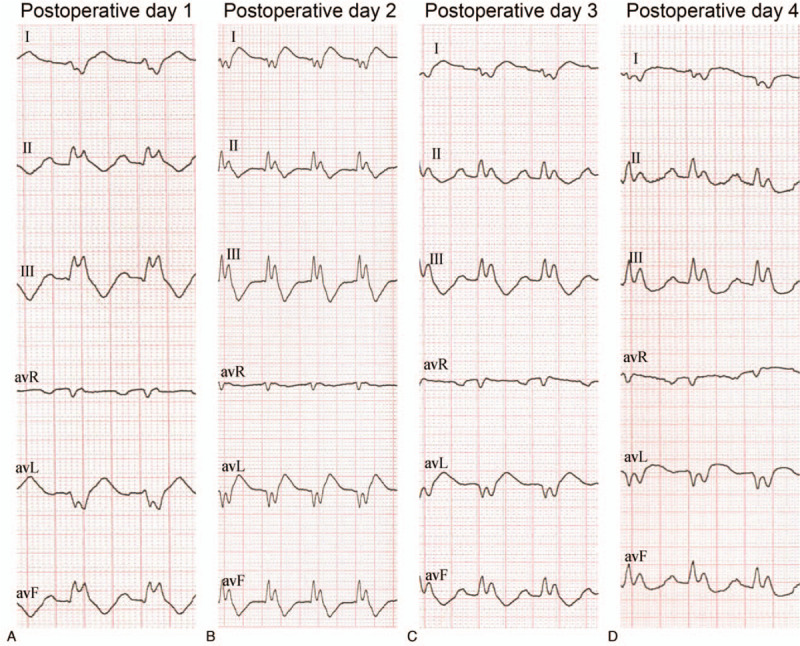
Dynamic echocardiography (ECG) changes of fragmented PRS complex postoperatively. (A) Postoperative day 1. (B) Postoperative day 2. (C) Postoperative day 3. (D) Postoperative day 4.

The postoperative chest X-ray exhibited signs of pulmonary edema and exudative lesions in the left middle lobe. UCG showed an ejection fraction (EF) of 59%. Blood results were as follows: N-terminal pro b-type natriuretic peptide (NT-proBNP) 2433 pg/mL, Lactate (Lac) 4.7 mmol/L, White blood cell count (WBC) 16.79∗109/L Neutrophil (N) 90.6%, Alaine aminotransferase (ALT) 213 U/L, serum creatinine (Scr) 146 umol/L, C-reactive protein (CRP) 17 mg/L, Creatine kinase-MB (CK-MB) 262.9 ng/mL, TnT > 10,000 pg/mL. Diuretics, vasopressor and antibiotics were administered to treat the heart failure and pulmonary infection. A new-onset persistent fragmented QRS complex with various RSR patterns with high-grade atrioventricular block indicated an acute inferior myocardial infarction (Fig. 4).

On postoperative day 3, the patient presented with a consistent sinus tachycardia (HR ∼130 bpm) with decreased central venous pressure. Fluid supplement was administered and the vasopressor was gradually withdrawn. The patient returned to a stable condition and was eventually transferred to the general ward.

**Figure 5:**
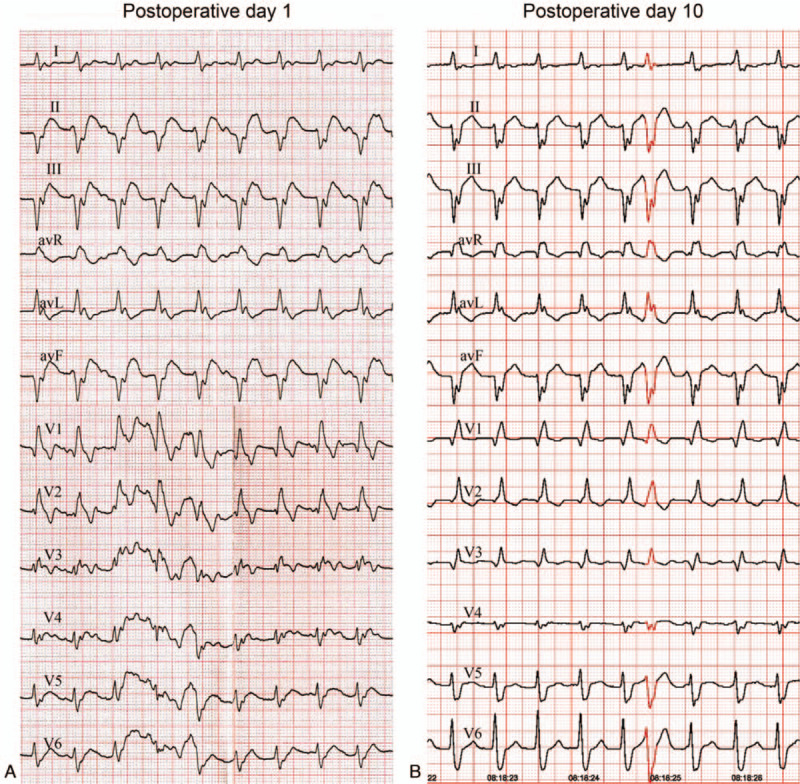
Echocardiography (ECG) displayed non-paroxysmal supraventricular tachycardia.

The 1st day after the operation, ECG showed sinus tachycardia, non-paroxysmal junctional tachycardia, atrioventricular dissociation with interference, LAFB, and CRBBB (Fig. 5A). On postoperative day 10, non-paroxysmal supraventricular tachycardia was still present on ECG (Fig. 5B). UCG showed an enlarged left ventricle, as well as decreased diastolic and systolic function with an EF of 40%. Beta-blocker was prescribed for long-term follow-up. Two weeks after the event, the elevated TnT and NT-proBNP levels fell to their lowest. A final definite diagnosis was made as coronary artery disease (CAD), acute anteroseptal and anterior ST-segment elevation myocardial infarction, acute inferior non-ST-segment elevation myocardial infarction, arrhythmia, high-grade atrioventricular block, LAFB, CRBBB, non-paroxysmal junctional tachycardial, atrioventricular dissociation with interference, cardiogenic shock, and Killip class IV.

## 3 Discussion

Acute ST-elevation myocardial infarction (STEMI) is caused by complete occlusion of a coronary artery leading to sudden perfusion insufficiency.^[8]^ STEMI with simultaneous multi-vessel coronary thrombosis, however, has been rarely reported. This combination may occur secondary to coronary vasospasm, cocaine abuse, or other unidentifiable causes.^[9,10]^

Only about 4.8% of STEMI cases concurrently present with thrombosis affecting >1 coronary artery.^[6]^ There are several characteristics of this rare condition. First, patients with STEMI and simultaneous thrombosis are primarily male (88%). Secondly, either RCA and LAD or RCA and LCX are the primary culprit coronary arteries with thrombus burden (50%). In the current case, thrombosis in LAD and LCX concurrently is rarely seen. Third, cardiogenic shock was the predominant presentation (41%). Fourth, IABP (38%) could be conducted due to an impairment of cardiac output. The patient's manifestations were inconsistent with all of these reported characteristics.^[4]^ It is noteworthy that the prevalence of this condition might be underestimated because the incidence of multiple plaques in the setting of STEMI has been reported to be 40% to 65% and thrombosis was evident in up to 50% of sudden death cases.^[11–14]^ Acute chest pain with new-onset multi-bundle branch block usually indicates extensive myocardial infarction, which requires prompt circulatory assistance. Simultaneous thrombosis in multiple branches, especially concurrent thrombosis in LAD and LCX, rarely occurs and is difficult to diagnose. The current case presented complex and variable arrhythmia, which is associated with cardiogenic shock and a high risk of mortality. Temporary pacing and IABP support were necessary and the patient required a long duration of hospital stay.

Changes in the ECG from a QS wave to an rS wave in the inferior wall indicated the formation of embryonic R wave suggesting an incomplete inferior myocardial infarction. The ECG findings were consistent with the CAG observation, which showed a 99% stenosis in the distal LCX with TIMI grade 2. A concurrent anterior and inferior myocardial infarction was confirmed by CAG. We speculate that acute anterior myocardial infarction formed due to atherosclerosis-induced acute artery occlusion. The impaired cardiac output precipitated the development of thrombosis in the circumflex branch which manifested as inferior myocardial infarction.

In addition to cardiogenic shock, different types of arrhythmias occurred in the case. The LAFB and anterior myocardial infarction intermittently appeared postoperatively. This finding implies that the presence of LAFB could mask anterior myocardial infarction. For patients with LAFB and typical manifestations, susceptible myocardial infarction should be considered. The patient also had dynamic alterations of a fragmented QRS complex including various RSR’ patterns in the inferior leads. Given that thrombosis was observed in the distal LCX, an inferior myocardial infarction was confirmed. Fragmented QRS indicates a prior myocardial infarction. The sensitivity is 91.4%, higher than that of pathological Q waves (36.3%). New-onset fragmented QRS has a specificity of 96% in the diagnosis of myocardial infarction^[15]^ and patients with new fragmented QRS have a significantly shorter survival than those without fragmented QRS.^[16]^ In this case, we speculated that fragmented QRS was a sign of acute inferior myocardial infarction because:

1. A new-onset fragmented wave was commonly seen within the first 48 h of myocardial infarction.
2. Previous studies demonstrated that fragmented waves associated with acute myocardial infarction had dynamic changes, which was consistent with the variable changes of ECG in this case.
3. Coronary arteriography showed acute thrombosis in the distal LCX.

Non-paroxysmal junctional tachycardia, a kind of arrhythmia arising from a discrete focus within the AV node or His bundle, is associated with automaticity or triggered activity instead of re-entry. It is not commonly seen in patients with myocardial infarction. Most cases occur during the first few days after myocardial infarction. As shown in a previous study, 40% of myocardial infarction patients experienced non-paroxysmal junctional tachycardia within the first 24 h, 13% in 24 to 48 h, and only 3% in 48 to 72 h.^[17]^ However, this case showed that non-paroxysmal junctional tachycardia could still develop even after 10 days. Treatment for symptomatic non-paroxysmal junctional tachycardia included beta-blockers, intravenous adenosine, or verapamil.^[18]^

IABP implantation improves myocardial oxygenation and increases cardiac output and organ perfusion with a reduction in left ventricular workload. However, optimum hemodynamic performance has only been achieved when IABP was performed with a HR of 80 to 110 bpm; HR > 110 bpm resulted in a reduced benefit of IABP.^[19]^

## 4 Conclusions

Acute chest pain with new-onset multi-bundle branch block usually indicates extensive myocardial infarction, which requires prompt circulatory assistance. Concurrent thrombosis in LAD and LCX rarely occurs and is hard to diagnose. The current case presented complex and variable arrhythmia associated with cardiogenic shock and a high risk of mortality. Temporary pacing and IABP support was crucial measurement for this patient. The inferior embryonic r wave may mask the pathological Q wave of inferior myocardial infarction and the left anterior branch block may mask the pathological Q wave of anterior and anteriorseptal myocardial infarction. In the event of new-onset fragmented QRS waves in patients with chest pain, clinicians should be alert. Non-paroxysmal junctional tachycardia could occur even after the first 72 h after myocardial infarction.

## Author contributions

Chuang-jia Hu and Mu-li Wu performed most of the investigation, data analysis and wrote the manuscript; Duan-Min Xu and Chang Chen provided pathological assistance; Ye-qun Chen and Yi-Fan Sun contributed to interpretation of the data and analyses. All of the authors have read and approved the manuscript.

**Conceptualization:** Mu-li Wu.

**Data curation:** Mu-li Wu, Yi-Fan Sun.

**Formal analysis:** Mu-li Wu.

**Funding acquisition:** Chang Chen, Ye-qun Chen, Yi-Fan Sun.

**Investigation:** Ye-qun Chen.

**Methodology:** Duan-Min Xu, Chang Chen.

**Resources:** Yi-Fan Sun.

**Software:** Ye-qun Chen.

**Supervision:** Duan-Min Xu, Ye-qun Chen.

**Validation:** Mu-li Wu, Duan-Min Xu.

**Visualization:** Mu-li Wu.

**Writing – original draft:** Chang Chen.

**Writing – review & editing:** Chang Chen.
